# Comparative Evaluation of Anti-HER2 Affibody Molecules Labeled with ^64^Cu Using NOTA and NODAGA

**DOI:** 10.1155/2017/8565802

**Published:** 2017-02-28

**Authors:** Vladimir Tolmachev, Cheng-Bin Yim, Johan Rajander, Anna Perols, Amelie Eriksson Karlström, Merja Haaparanta-Solin, Tove J. Grönroos, Olof Solin, Anna Orlova

**Affiliations:** ^1^Department of Immunology, Genetics and Pathology, Uppsala University, 75185 Uppsala, Sweden; ^2^Turku PET Centre, University of Turku, P.O. Box 52, 20521 Turku, Finland; ^3^Turku PET Centre, Åbo Akademi University, P.O. Box 52, 20521 Turku, Finland; ^4^Division of Protein Technology, School of Biotechnology, KTH Royal Institute of Technology, 106 91 Stockholm, Sweden; ^5^MediCity Research Laboratory, University of Turku, 20520 Turku, Finland; ^6^Department of Oncology and Radiotherapy, Turku University Hospital, 20520 Turku, Finland; ^7^Department of Chemistry, University of Turku, 20014 Turku, Finland; ^8^Division of Molecular Imaging, Department of Medicinal Chemistry, Uppsala University, 751 05 Uppsala, Sweden

## Abstract

Imaging using affibody molecules enables discrimination between breast cancer metastases with high and low expression of HER2, making appropriate therapy selection possible. This study aimed to evaluate if the longer half-life of ^64^Cu (*T*_1/2_ = 12.7 h) would make ^64^Cu a superior nuclide compared to ^68^Ga for PET imaging of HER2 expression using affibody molecules. The synthetic ZHER2:S1 affibody molecule was conjugated with the chelators NOTA or NODAGA and labeled with ^64^Cu. The tumor-targeting properties of ^64^Cu-NOTA-ZHER2:S1 and ^64^Cu-NODAGA-ZHER2:S1 were evaluated and compared with the targeting properties of ^68^Ga-NODAGA-ZHER2:S1 in mice. Both ^64^Cu-NOTA-ZHER2:S1 and ^64^Cu-NODAGA-ZHER2:S1 demonstrated specific targeting of HER2-expressing xenografts. At 2 h after injection of ^64^Cu-NOTA-ZHER2:S1, ^64^Cu-NODAGA-ZHER2:S1, and ^68^Ga-NODAGA-ZHER2:S1, tumor uptakes did not differ significantly. Renal uptake of ^64^Cu-labeled conjugates was dramatically reduced at 6 and 24 h after injection. Notably, radioactivity uptake concomitantly increased in blood, lung, liver, spleen, and intestines, which resulted in decreased tumor-to-organ ratios compared to 2 h postinjection. Organ uptake was lower for ^64^Cu-NODAGA-ZHER2:S1. The most probable explanation for this biodistribution pattern was the release and redistribution of renal radiometabolites. In conclusion, monoamide derivatives of NOTA and NODAGA may be suboptimal chelators for radiocopper labeling of anti-HER2 affibody molecules and, possibly, other scaffold proteins with high renal uptake.

## 1. Introduction

Treatment of disseminated breast cancer, which overexpresses human epidermal growth factor receptor type 2 (HER2), with the monoclonal antibody trastuzumab, the antibody-drug conjugate trastuzumab DM-1, or the tyrosine kinase inhibitor lapatinib improves the survival of patients [1]. HER2 overexpression is a predictive biomarker for HER2-targeting therapies [2]. The use of sensitive radionuclide molecular imaging may permit repetitive noninvasive assessment of HER2 expression in breast cancer metastases, addressing the issue of spatial and temporal heterogeneity of HER2 expression. The use of radiolabeled HER2-specific antibodies [3, 4] and their fragments [5] for clinical imaging of HER2 expression has been reported.

Mathematic modeling suggests that for a proteinaceous imaging probe the combination of small size with high affinity provides the highest contrast and consequently the highest sensitivity of radionuclide molecular imaging [6]. Indeed, excellent contrast was demonstrated in a clinical study that used the smallest possible antibody fragment (VHH) [7]. It is possible to develop even smaller targeting probes by using engineered nonimmunoglobulin scaffold proteins [8], which can be 2- to 4-fold smaller than VHH fragments. Their affinity to selected targets may be in the single-digit nanomolar or subnanomolar range. Other potential advantages of nonimmunoglobulin scaffold proteins include the possibility of their production in prokaryotic hosts or by peptide synthesis, which would dramatically reduce production costs. Moreover, they are structurally stable and can refold after denaturation, permitting the use of harsh labeling conditions. Preclinical studies demonstrate the successful application of such scaffold proteins as targeting probes for radionuclide molecular imaging, including DARPins [9], knottins [10], ADAPTs [11], fibronectin domains [12], and affibody molecules [13]. Clinical studies show that ^68^Ga-labeled affibody molecules enable high-contrast imaging of HER2 expression in tumors, can discriminate between breast cancer metastases with high and low HER2 expression, are nontoxic and nonimmunogenic, and inflict a low absorbed dose burden in patients [14, 15].

Clinical PET studies using anti-HER2 affibody molecules labeled with ^68^Ga (*T*_1/2_ = 67.9 min) demonstrated that discrimination between high and low HER2 expression is better at 4 h postinjection (p.i.) than at 2 h p.i. [14]. This is agreement with preclinical [16] and clinical [17] studies demonstrating that retention of affibody-associated radioactivity is better in tumors with higher HER2 expression compared to tumors with low expression. However, the short half-life of ^68^Ga complicates imaging later than 4 h after injection. Thus, it would be advantageous to use a positron-emitting label with a longer half-life than the half-life of ^68^Ga. Candidate labels that could provide imaging several hours after injection include two positron-emitting copper radioisotopes: ^61^Cu (*T*_1/2_ = 3.4 h) and ^64^Cu (*T*_1/2_ = 12.7 h). Both radionuclides can be produced using low-energy cyclotrons available at PET centres [18, 19].

The development of a radiocopper-labeled tracer requires the determination of an appropriate chelator. Although the macrocyclic tetraaza chelator DOTA provides thermodynamically stable and kinetically inert complexes with a variety of radiometals [20], a DOTA-complex of copper (II) is unstable in vivo. Released radiocopper can bind blood proteins and superoxide dismutase in the liver, deteriorating the imaging contrast [21]. This has been observed also in preclinical studies using affibody molecules and their derivatives. For example, the ZHER2:477 affibody molecule labeled with ^64^Cu using maleimido-DOTA-conjugated at the C-terminus had a hepatic uptake of 7.1 ± 1.7% ID/g at 4 h p.i. and 10.4 ± 1.2% ID/g at 20 h p.i. in a murine model (Balb/C nu/nu) [22]. When the same clone (designated as PEP05838) was labeled with ^111^In using the same chelator [16], the hepatic uptake was much lower, 2.1 ± 0.5% ID/g at 4 h p.i. and 1.7 ± 0.3% ID/g at 24 h p.i., in the same murine model. There was no clearance of radionuclide from blood between 4 h (0.52 ± 0.22% ID/g) and 20 h (0.71 ± 0.02% ID/g) in the case of the ^64^Cu label [22]. In the case of the ^111^In label, the bloodborne radioactivity was reduced from 0.13 ± 0.02% ID/g at 4 h p.i to 0.07 ± 0.01% ID/g at 4 h p.i. [16]. In another study, Ren and coworkers [23] compared the biodistribution of an ^111^In- and ^64^Cu-labeled DOTA-conjugated 2-helix variant of the anti-HER2 affibody molecule DOTA–MUT-DS. At 1 h after injection, the hepatic uptake of ^64^Cu-DOTA–MUT-DS was 28 ± 6% ID/g, while the hepatic uptake of ^111^In- DOTA–MUT-DS was 10 ± 2% ID/g, that is, nearly threefold lower. Clearing of radioactivity from blood was very slow in the case of ^64^Cu-DOTA–MUT-DS, from 1.5 ± 0.2% ID/g at 1 h p.i. to 1.10 ± 0.02% ID/g at 20 h p.i. In the case of ^111^In-DOTA–MUT-DS, the blood clearance was much more rapid, from 1.1 ± 0.1% ID/g at 1 h p.i. to 0.18 ± 0.02% ID/g at 20 h p.i. The results of the preclinical studies suggested that the triaza chelators NOTA and NODAGA produce in vivo stable complexes with radiocopper. These chelators and their derivatives have been successfully used to label a number of peptides and antibody fragments with copper-64 [24–27]. Adding to the importance of chelator selection, it has been shown that the charge and geometry of the radiometal-chelator complexes influence the off-target interactions of affibody molecules, modifying biodistribution and imaging contrast [28–31]. To date, ^68^Ga-NODAGA-ZHER2:S1 provides the best contrast among the tested ^68^Ga-labeled synthetic affibody molecules in preclinical studies [29].

**Figure 1:**
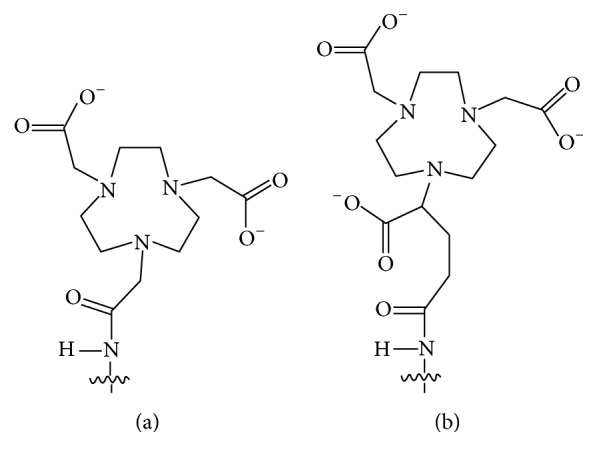
Structures of the NOTA (a) and NODAGA (b) chelators conjugated via an amide bond to the N-terminus of affibody molecules.

The present study aimed to evaluate the tumor-targeting and imaging properties of the synthetic affibody molecule ZHER2:S1 labeled with ^64^Cu using the NOTA and NODAGA chelators (Figure 1). We further compared their properties with the properties of ^68^Ga-NODAGA-ZHER2:S1, the best currently available ^68^Ga-labeled variant.

## 2. Materials and Methods

### 2.1. Measurements and Analysis

In the in vitro experiments and ex vivo animal studies, radioactivity uptake was measured using the Wizard^2^ automated gamma-counter (PerkinElmer). Formulation was accomplished using a VDC-405 dose calibrator (Veenstra Instruments). Radiochemical yield and purity were determined by radio-instant thin-layer chromatography (radio-ITLC) using ITLC-SG strips (Agilent), with elution in 0.2 M citric acid, pH 2. The ITLC strips were dried and exposed to BAS-TR2025 imaging plates (Fuji Photo Film Co.). To obtain digital images, we scanned the imaging plates with an FLA-5100 scanner (Fuji Photo Film Co). The images were analyzed using AIDA analysis software, version 4 (Raytest). Radio-ITLC results were cross-validated using radio-HPLC with a Jupiter Proteo C12 column (4.6 × 250 mm), at a flow rate of 1.0 mL/min, with a linear CH_3_CN/H_2_O gradient (10–70% CH_3_CN in 12 min) in 0.1% trifluoroacetic acid solution. The radio-HPLC data were concordant with the data from radio-ITLC.

The results are presented as mean ± standard deviation (SD). A *P* value of <0.05 (based on unpaired two-tailed *t*-test) was considered to indicate a significant difference between two groups. To evaluate differences between more than two groups, we performed one-way ANOVA analysis with Bonferroni's multiple comparison test using Prism 5 software (GraphPad Software).

### 2.2. Production of ^64^Cu

High quality water was deionized (resistance higher than 18 MΩ/cm^2^) by passing it through the Ultra Clear filtration system (SG Wasseraufbereitung und Regenerierstation GmbH, Germany). Ultrapure grade aqueous acids (Carl Roth GmbH, Germany) with ppt-levels of metal impurities were used for all solutions during ^64^Ni-electrodeposition, Cu/Ni-separation, and ^64^Cu-formulation. ^64^Cu in the form of [^64^Cu]CuCl_2_ was produced via the ^64^Ni(p,n)^64^Cu nuclear reaction, as previously described [18, 32]. In brief, ^64^Cu is produced by proton bombardment of an enriched ^64^Ni target (>98%, 80 mg) with proton energy of 13 MeV using a CC-18/9 cyclotron (D.V. Efremov Institute, St. Petersburg, Russia). Radiochemical isolation of ^64^Cu by anion exchange chromatography and recovery of ^64^Ni were performed as previously described [33, 34]. ^64^Cu was formulated as 16–18 MBq/*μ*L in 0.04 M HCl-solution. The effective specific radioactivity (ESA) was at least 3 TBq/*μ*mol at the end of bombardment (EoB), as determined by titration with 1,4,8,11-tetraazacyclotetradecane-1,4,8,11-tetraacetic acid (TETA) (Sigma-Aldrich, Germany).

### 2.3. Labeling and Stability

We synthetically produced, purified, and characterized affibody molecules containing NOTA or NODAGA chelators coupled via an amide bond to an N-terminal alanine as previously described [28]. Both variants showed a purity of over 97%.

Labeling was performed using two different protocols. In Protocol A, affibody molecules (50 *μ*g) were reconstituted in 50 *μ*L 0.55 M ammonium acetate, pH 5.6, and mixed with 150 MBq ^64^CuCl_2_ in 10 *μ*L 0.04 M HCl. This mixture was incubated for 45 min at 95°C and then diluted with PBS and analyzed. In Protocol B, following incubation with ^64^CuCl_2_, the reaction mixture was combined with a 500-fold excess of Na_4_EDTA (10 mg/mL in water, 137 *μ*L). This mixture was then incubated at 95°C for an additional 10 min, and radiolabeled affibody molecules were purified using disposable NAP-5 size-exclusion columns (GE Healthcare).

To test the labeling stability, samples of radiolabeled affibody molecules were incubated for two hours at room temperature with a 500-fold excess of Na_4_EDTA. Control samples were diluted with the same volume of PBS. Radio-ITLC was used to measure the percentage of protein-bound radioactivity.

For comparative studies NODAGA-ZHER2:S1 was labeled with ^68^Ga as previously described [29]. For in vitro displacement experiments, the anti-HER2 affibody molecule ZHER2:2395 was labeled with ^99m^Tc as previously described [35]. Loading of NODAGA-ZHER2:S1 with ^nat^Cu and ^nat^Ga and of NOTA-ZHER2:S1 with ^nat^Cu was performed following the exact same protocols as for labeling with a fivefold molar excess of metals over proteins.

### 2.4. Binding to and Processing by HER2-Expressing Cells In Vitro

The HER2-expressing SKOV-3 ovarian carcinoma cell line (American Type Culture Collection, ATCC) was used for binding specificity and cellular processing studies. DMEM medium (Lonza, Belgium) supplemented with 10% fetal bovine serum and penicillin (100 U/mL)–streptomycin (100 *μ*g/mL) (both from Biochrom AG) was used for culturing. For binding and cellular processing experiments, the cells were seeded one day before the experiment in Petri dishes. At the day of the experiment there were approximately 10^6^ cells/dish.

To test the specific binding of ^64^Cu-NODAGA-ZHER2:S1 and ^64^Cu-NOTA-ZHER2:S1 to HER2-expressing SKOV-3 cells, we performed a saturation assay [36] using a 100-fold molar excess of nonlabeled affibody molecules. The binding strengths of Cu-NODAGA-ZHER2:S1, Ga-NODAGA-ZHER2:S1, and Cu-NOTA-ZHER2:S1 were compared via measurement of their concentrations at half-maximum inhibition of ^99m^Tc-ZHER2:2395 binding to SKOV-3 cells (IC_50_), as previously described [30]. Briefly, SKOV-3 cells were incubated for 4 h at 4°C with ^99m^Tc-ZHER2:2395 (1 nM) in the presence of gallium- or copper-loaded affibody molecules (concentration range 0–500 nM). After incubation, the cells were washed with 3 mL of medium and treated with 1 mL of trypsin-EDTA solution. The detached cells were collected, and the cell-associated radioactivity was measured. The IC_50_ values were determined using GraphPad Prism software.

We used a previously validated modified acid wash method for processing of bound conjugates by HER2-expressing SKOV-3 cells [36]. Briefly, the cells were incubated with the labeled compound (1 nM) at 37°C. At predetermined time points, the medium from a set of three dishes was removed. The cells were washed twice with 1 mL of ice-cold medium. To collect the membrane-bound radioactivity, the cells were treated with 0.5 mL of 0.2 M glycine buffer containing 4 M urea, pH 2.0, for 5 min on ice. Dishes were additionally washed with 0.5 mL acidic buffer followed by 1 mL PBS, and the fractions were pooled. To collect radioactivity internalized by the cells, we treated them with 0.5 mL of 1 M NaOH at 37°C for 0.5 h. The dishes were additionally washed with 0.5 mL NaOH solution followed by 1 mL PBS, and the alkaline fractions were pooled and the percentage of internalized radioactivity was calculated.

### 2.5. Biodistribution Studies

Animals were cared for in compliance with the guidelines of the International Council of Laboratory Animal Science. All animal procedures were approved by the Animal Ethics Committee of the Provincial Government of Southern Finland and performed following the guidelines of the European Community Council Directives 86/609/EEC.

Female BALB/C nu/nu mice (Scanbur, 8 weeks old at arrival) were used to obtain HER2-positive tumors by subcutaneous inoculation of 1 × 10^7^ SKOV-3 cells (*n* = 28) or HER2-negative control tumors by inoculation of 1 × 10^7^ Ramos lymphoma cells (*n* = 8). Experiments were performed 26 days after implantation. The average tumor weight was 0.14 ± 0.1 g at the time of the experiment.

For ex vivo measurements, we used a group of four mice for each tracer and data point. Mice bearing SKOV-3 xenografts were injected with ^64^Cu-NODAGA-ZHER2:S1 or ^64^Cu-NOTA-ZHER2:S1 (450 kBq per mouse) via the tail vein. The injected protein dose was adjusted to 5 *μ*g (0.7 nmol). The dose was selected based on our data showing that variation of the injected dose of anti-HER2 affibody molecules between 1 and 10 *μ*g per mouse has no significant effect on the uptake in SKOV-3 xenografts [37]. Biodistribution was measured 2, 6, and 24 h postinjection. Mice bearing Ramos xenografts were injected with the same amounts of protein and radioactivity, and biodistribution was measured 2 h after injection. Similarly, ^68^Ga-NODAGA-ZHER2:S1 biodistribution was measured 2 h after injection of 5 *μ*g/500 kBq per mouse. Three mice were injected with ^64^Cu-citrate (450 kBq per mouse), and radioactivity distribution was evaluated 2 h postinjection. The citrate was used to prevent rapid formation of nonsoluble hydroxide. For ex vivo tissue distribution measurements, the animals were anesthetized and exsanguinated, and the organs of interest were excised. Lung, liver, spleen, stomach wall, and kidneys were sampled as whole organs. Besides, radioactivity of gastrointestinal tract (with content) and carcass was measured.

### 2.6. In Vivo Imaging Studies

SKOV-3 (*n* = 2) or Ramos (*n* = 2) xenograft-bearing mice were injected via the tail vein with ^64^Cu-NODAGA-ZHER2:S1 (9.7 ± 1.4 MBq, 5 *μ*g, 120 *μ*L) and ^64^Cu-NOTA-ZHER2:S1 (10.5 ± 0.9 MBq, 5 *μ*g, 120 *μ*L). Additionally, SKOV-3 (*n* = 2) xenograft-bearing mice were injected with ^68^Ga-NODAGA-ZHER2:S1 (2.3 ± 0.1 MBq, 5 *μ*g, 120 *μ*L). Mice were anesthetized using 2.5% isoflurane/O_2_ and positioned on a heating pad two at a time in a PET/CT scanner (Siemens Medical Solutions, Inc.) for CT acquisition (10 min) and PET scan in list mode (20 min). Mice bearing SKOV-3 xenografts were scanned at 2, 6, and 24 h postinjection and mice with Ramos xenografts at 2 h postinjection. PET images were reconstructed using an FBP algorithm of two iterations, followed by maximum* a posteriori* (MAP, 18 iterations) integrative algorithms (Inveon Acquisition Workplace, version 2.0; Siemens Preclinical Solutions). Data were decay-corrected to the time of injection.

## 3. Results

### 3.1. Labeling and Stability

Labeling of both NOTA-ZHER2:S1 and NODAGA-ZHER2:S1 with ^64^Cu was performed in 0.55 M ammonium acetate, pH 5.6, using two different protocols.  Table 1 presents the results of the ^64^Cu-labeling experiments and the stability tests. Labeling using Protocol A led to incorporation of >95% of ^64^Cu into the affibody molecules. However, about 6% of the radioactivity was released upon EDTA challenge. We hypothesized that a fraction of the copper was not stably complexed by a macrocyclic chelator but was instead loosely bound to a weak “chelator pocket” formed by amino acids. Protocol B included an EDTA challenging step before purification to strip this weakly bound radiometal. This additional step decreased the overall yield by about 15% but produced conjugates that could withstand the EDTA challenge. The purity was over 98% for both the ^64^Cu-labeled NOTA and NODAGA conjugates. Specific activity of 2.5 MBq/*μ*g (17.4 GBq/*μ*mol) was obtained. Due to better stability of the label, Protocol B was used in biological studies.

### 3.2. Binding to and Processing by HER2-Expressing Cells In Vitro

**Figure 2:**
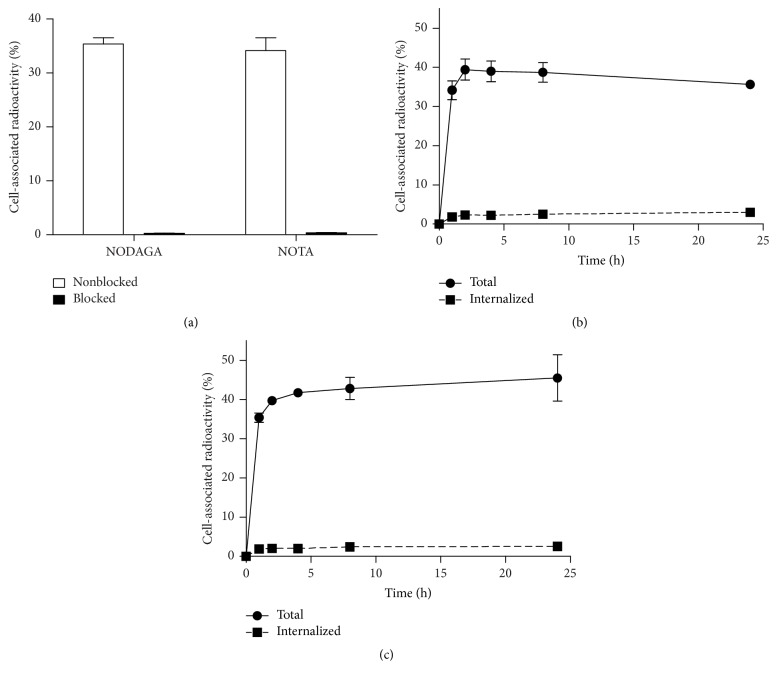
(a) In vitro binding specificity of ^64^Cu-NODAGA-ZHER2:S1 and ^64^Cu-NOTA-ZHER2:S1 to HER2-expressing SKOV-3 cells. In the blocked group, receptors were presaturated with a 100-fold excess of nonlabeled affibody molecules. Panels (b) and (c) show the cellular processing of ^64^Cu-NOTA-ZHER2:S1 (b) and ^64^Cu-NODAGA-ZHER2:S1 (c) by SKOV-3 cells. Cells were incubated with the conjugate (1 nM) at 37°C. Data are presented as the mean of three samples ± SD.

Figure 2(a) presents the results of the specificity test. Presaturation of receptors with nonlabeled counterparts significantly reduced (*P* < 0.00005) the binding of both ^64^Cu-NODAGA-ZHER2:S1 and ^64^Cu-NOTA-ZHER2:S1 to HER2-expressing cells, demonstrating the HER2-specificity of both radioligands.

Figures 2(b) and 2(c) present the cellular processing of ^64^Cu-NODAGA-ZHER2:S1 and ^64^Cu-NOTA-ZHER2:S1. Both conjugates showed a low fraction of internalized radioactivity, with less than 10% of cell-associated radioactivity detected at 24 h after incubation. The two conjugates differed somewhat in their overall uptake patterns, with ^64^Cu-NODAGA-ZHER2:S1 binding showing an ascending tendency and ^64^Cu-NOTA-ZHER2:S1 binding showing a descending tendency after 2 h. Total cell-associated activity at 24 h was significantly higher (*P* < 0.05) for ^64^Cu-NODAGA-ZHER2:S1 than for ^64^Cu-NOTA-ZHER2:S1.

**Figure 3:**
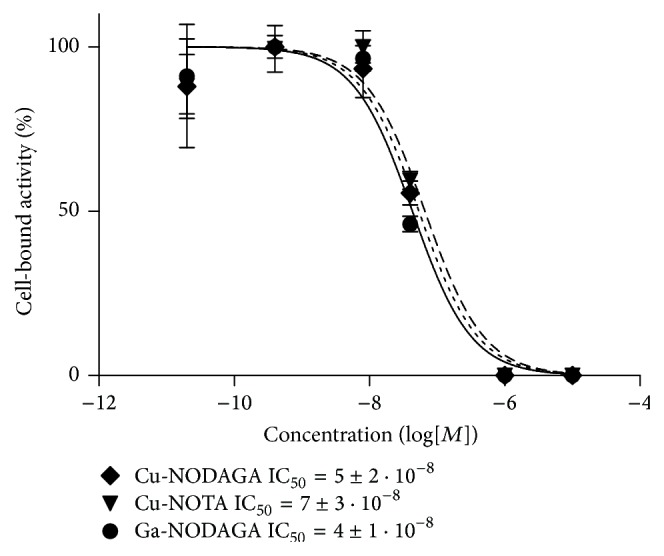
Inhibition of ^99m^Tc-ZHER2:2395 binding to SKOV-3 cells with ^nat^Cu-NODAGA-ZHER2:S1, ^nat^Ga-NODAGA-ZHER2:S1, or ^nat^Cu-NOTA-ZHER2:S1. The data are presented as mean ± SD of three samples.

The relative binding strengths of Cu-NODAGA-ZHER2:S1, Ga-NODAGA-ZHER2:S1, and Cu-NOTA-ZHER2:S1 were compared via measurement of their concentrations at half-maximum inhibition of ^99m^Tc-ZHER2:2395 binding to SKOV-3 cells (IC_50_). The IC_50_ values did not significantly differ between ^nat^Cu-NODAGA-ZHER2:342, ^nat^Ga-NODAGA-ZHER2:S1, and ^nat^Cu-NOTA-ZHER2:S1 (Figure 3), suggesting that neither chelators nor metals (in the case of Ga-NODAGA-ZHER2:342) affected the binding strength of ZHER2:S1 to HER2-expressing cells.

### 3.3. Biodistribution Studies

**Figure 4:**
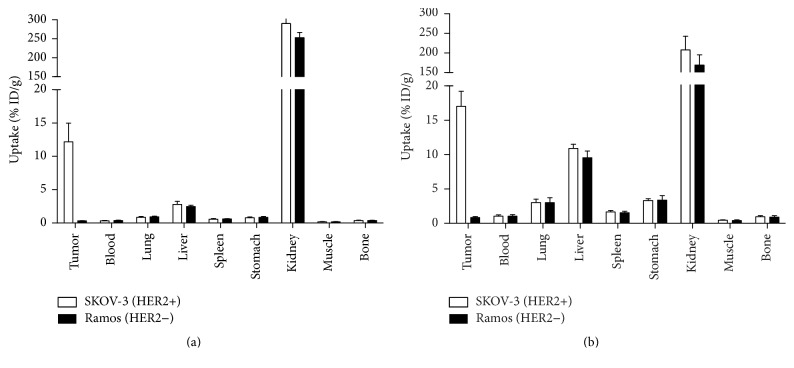
Uptake of ^64^Cu-NODAGA-ZHER2:S1 (a) or ^64^Cu-NOTA-ZHER2:S1 (b) at 2 h after injection in mice bearing either HER2-positive xenografts (SKOV-3) or HER2-negative xenografts (Ramos). The data are presented as mean ± SD for four mice.

The tumor-targeting properties of the affibody molecules were compared in BALB/C nu/nu mice bearing implanted human cancer xenografts. To confirm targeting specificity in vivo, we evaluated ^64^Cu-NOTA-ZHER2:S1 and ^64^Cu-NODAGA-ZHER2:S1 uptake in HER2-positive SKOV-3 xenografts versus HER2-negative Ramos xenografts at 2 h after injection (Figure 4). The highly significant difference (*P* < 0.0005) between uptakes in HER2-positive and HER2-negative xenografts at 2 h postinjection confirmed the in vivo targeting specificity. The uptake of these tracers did not differ significantly in any other tissue of mice bearing HER2-positive and HER2-negative xenografts.

**Figure 5:**
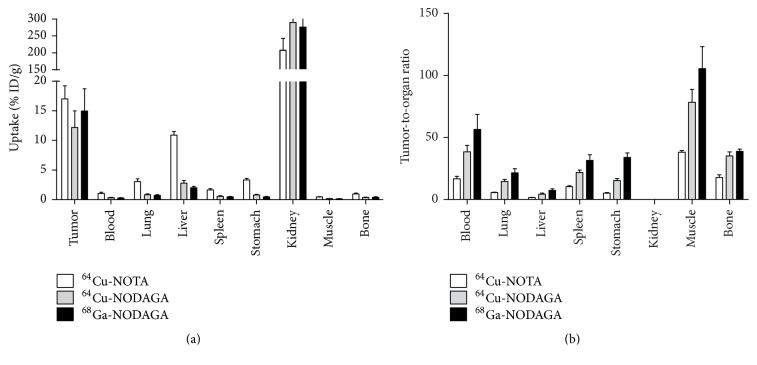
Comparison of ^64^Cu-NOTA-ZHER2:S1, ^64^Cu-NODAGA-ZHER2:S1, and ^68^Ga-NODAGA-ZHER2:S1 biodistribution (a) and tumor-to-organ ratios (b) at 2 h after injection in mice bearing HER2-positive SKOV-3 xenografts. The data are presented as mean ± SD for four mice.

Figure 5(a) presents a comparison of the biodistributions of ^64^Cu-NOTA-ZHER2:S1, ^64^Cu-NODAGA-ZHER2:S1, and ^68^Ga-NOADGA-ZHER2:S1 at 2 h postinjection in mice bearing HER2-expressing SKOV-3 xenografts. As is typical for affibody molecules, cleared tracers were reabsorbed in the kidneys. At that time point, radioactivity was localized in the tumors (with no significant difference between the conjugates, *P* > 0.017) and cleared from other normal organs and tissues. Uptake did not significantly differ between ^64^Cu-NODAGA-ZHER2:S1 and ^68^Ga-NODAGA-ZHER2:S1 in any organ (*P* > 0.017). In contrast, ^64^Cu-NOTA-ZHER2:S1 uptake was significantly higher (*P* < 0.017) than that of ^64^Cu- and ^68^Ga-NODAGA-ZHER2:S1 in all organs, except the kidneys. Renal uptake was significantly lower for ^64^Cu-NOTA-ZHER2:S1 (*P* < 0.017). Compared to the other two tracers, ^64^Cu-NOTA-ZHER2:S1 showed significantly (*P* < 0.017) lower tumor-to-organ ratios (Figure 5(b)). Compared to both ^64^Cu-labeled variants, ^68^Ga-NODAGA-ZHER2:S1 provided significantly higher tumor-to-organ ratios (*P* < 0.017), with the exception of the tumor-to-bone and tumor-to-kidney ratios.

Table 2 presents biodistribution data for ^64^Cu-NOTA-ZHER2:S1 and ^64^Cu-NODAGA-ZHER2:S1 in mice bearing HER2-positive SKOV-3 xenografts at 2, 6, and 24 h after injection. Renal radioactivity levels rapidly decreased, which is unusual for radiometal-labeled affibody molecules. From 2 to 24 h postinjection, renal radioactivity decreased nearly 2.6-fold for ^64^Cu-NODAGA-ZHER2:S1 and nearly 15-fold for ^64^Cu-NOTA-ZHER2:S1. Uptake of ^64^Cu-NOTA-ZHER2:S1 in the blood, lung, liver, spleen, stomach, and gastrointestinal tract increased rapidly, peaking at 6 h postinjection. Uptake of ^64^Cu-NODAGA-ZHER2:S1 in the blood, lung, liver, spleen, and stomach gradually increased over time. Tumor uptake of ^64^Cu-NODAGA-ZHER2:S1 did not significantly differ between the 2, 6, and 24 h postinjection time points. On the other hand, tumor uptake of its NOTA-conjugated counterpart was significantly lower at 24 h compared to at 2 and 6 h. Accordingly, the tumor-to-organ ratios for both ^64^Cu-labeled conjugates decreased over time (Table 3). The tumor-to-organ ratios for ^64^Cu-NODAGA-ZHER2:S1 were significantly higher than those for ^64^Cu-NOTA-ZHER2:S1 at all time points.

Table 4 presents the biodistribution of free radiocopper (^64^Cu-citrate) at 2 h postinjection. Total body retention of radioactivity was 92 ± 5% ID, and blood radioactivity remained at the level of 2.1 ± 0.1% ID/g. Radioactivity accumulated in the lung, liver, spleen, and stomach organs, which all showed prominently increased radioactivity at 6 and 24 h after injection of ^64^Cu-NOTA-ZHER2:S1 and ^64^Cu-NODAGA-ZHER2:S1.

### 3.4. In Vivo PET Studies

**Figure 6:**
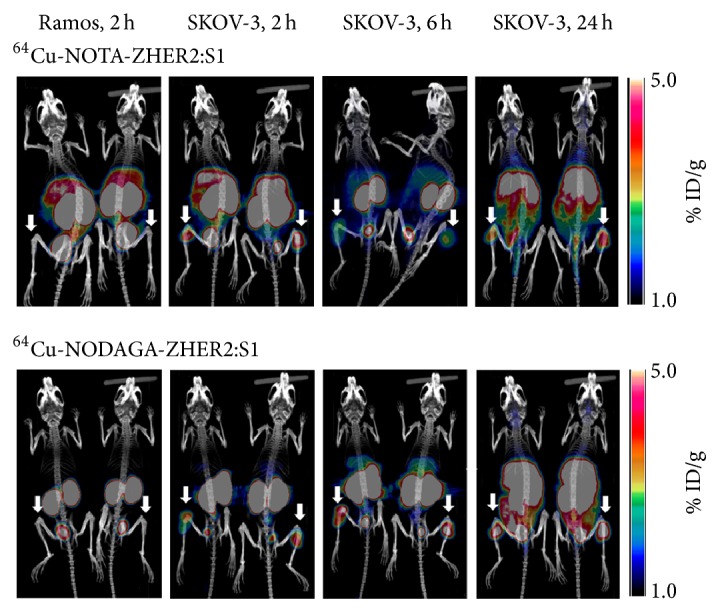
Maximum-intensity-projection PET/CT images of ^64^Cu-NOTA-ZHER2:S1 and ^64^Cu-NODAGA-ZHER2:S1 at 2, 6, and 24 h after injection in mice bearing HER2-positive xenografts (SKOV-3) and HER2-negative xenografts (Ramos). Arrows point at tumors.

**Figure 7:**
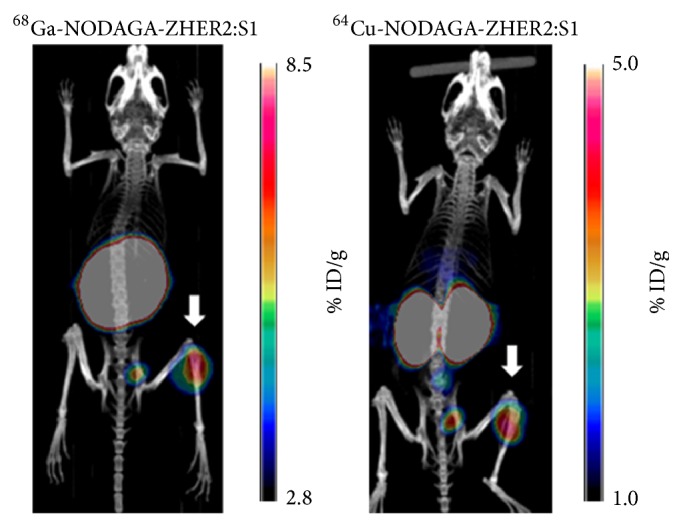
Representative maximum-intensity-projection PET/CT images of mice bearing HER2-positive SKOV-3 xenografts. Images were achieved by static scan at 2 h after the injection of ^68^Ga-NODAGA-ZHER2:S1 or ^64^Cu-NODAGA-ZHER2:S. Arrows point at tumors.

The biodistribution data were supported by our results from the imaging experiments (Figures 6 and 7). HER2-positive SKOV-3 xenografts were clearly visualized at all time points using radiocopper-labeled affibody molecules. The uptake of both tracers was much higher in HER2-positive xenografts than in HER2-negative xenografts. At all time points, the hepatic uptake of ^64^Cu-NODAGA-ZHER2:S1 was appreciably lower than the uptake of ^64^Cu-NOTA-ZHER2:S1. The uptake of ^64^Cu-NOTA-ZHER2:S1 over time was clearly decreased in the kidneys and increased in the liver. ^64^Cu-NODAGA-ZHER2:S1 and ^68^Ga-NODAGA-ZHER2:S1 enabled nearly equal visualization of SKOV-3 xenografts (Figure 7).

## 4. Discussion

The results of this study demonstrated that the macrocyclic chelators NOTA and NODAGA enabled efficient radiocopper labeling of the synthetic anti-HER2 affibody molecule ZHER2:S1 (Table 1). The inclusion of an EDTA challenge before final purification solved the issue of radiocopper being loosely bound to protein. Despite the harsh labeling conditions, radiocopper-labeled affibody molecules specifically bound to HER2-expressing cells in vitro (Figure 2(a)). The chelator-radionuclide combination did not significantly influence the strength of binding to SKOV-3 cells (Figure 3). Internalization of anti-HER2 affibody molecules is typically quite modest [18, 29, 36], but the internalized fractions of ^64^Cu-NOTA-ZHER2:S1 and ^64^Cu-NODAGA-ZHER2:S1 were unusually small, being two- to threefold lower than the internalized fractions of their ^68^Ga- and ^111^In-labeled counterparts [18, 29, 36] (Figure 2(b)). This may have been due to a slower internalization rate or, more likely, because of the moderate residualizing properties of the ^64^Cu-NOTA and ^64^Cu-NODAGA labels and the release of the radiometabolites following intracellular degradation.

Both ^64^Cu-NOTA-ZHER2:S1 and ^64^Cu-NODAGA-ZHER2:S1 demonstrated specific targeting and imaging of HER2-expressing xenografts in vivo (Figures 4 and 6). Interestingly, ^64^Cu-NODAGA-ZHER2:S1 and ^68^Ga-NODAGA-ZHER2:S1 showed very similar biodistribution at 2 h after injection, although the tumor-to-organ ratios were somewhat lower with ^64^Cu-NODAGA-ZHER2:S1 (Figure 5). However, ^64^Cu-NOTA-ZHER2:S1 administration resulted in substantially higher radioactivity levels in the majority of organs (Figure 5). This effect is apparently due to the use of the NOTA chelator. The amino-conjugated NOTA complex with copper (II) is neutral, while the complex with NODAGA is negatively charged. Earlier studies show that increasing the negative charge at the N-terminus of affibody molecules decreases the hepatic uptake and facilitates clearance from blood [18, 29, 38]. However, ^64^Cu-NOTA-ZHER2:S1 showed an almost 4-fold increase in hepatic uptake compared to its NODAGA-containing counterpart, which is discordant behavior compared to what might be expected based on previous studies with ^68^Ga- or ^111^In-labeled variants.

Insight into the nature of this phenomenon might be gained by analyzing the radioactivity distribution at several time points after the injection of radiocopper-labeled affibody molecules (Table 2). The typical biodistribution pattern for radiometal-labeled affibody molecules includes very slow release of radioactivity from tumors, liver, and kidneys, with appreciably more rapid clearance from blood, lung, and intestines, thus, increasing the tumor-to-organ ratios over time [13, 28, 39]. A different pattern was observed with the ^64^Cu-labeled affibody molecules. Although tumor radioactivity was sufficiently retained, there was a disproportionately rapid decrease of renal radioactivity along with increased uptake in the liver, blood, lung, and intestines (Table 2). These organs that showed elevated uptake also accumulated free copper (Table 4). Radiocopper could not have been directly released from the conjugates in blood/extracellular space, since the conjugation was stable (Table 1), the ^64^Cu complex with monoamide-NOTA is stable in blood [40], and no free conjugate was available to increase the radioactivity in all tissues except the kidneys. Thus, the unusual biodistribution of ^64^Cu-NOTA-ZHER2:S1 and ^64^Cu-NODAGA-ZHER2:S1 was most likely due to radiocatabolite release from the kidneys. This explanation correlates well with the poor intracellular retention of copper (Figure 2).

Importantly, the internalization of affibody molecules by HER2-expressing cells occurs slowly, while such internalization by proximal tubules occurs rapidly [41]. Compared to its complex with monoamide-NODAGA, the complex of ^64^Cu with monoamide-NOTA is apparently less stable under the conditions of the lysosomal compartment of the renal tubules. Interestingly, earlier publications have not highlighted the phenomenon of ^64^Cu “leakage” after the processing of complexes with NOTA and NODAGA. However, the prior studies were performed mainly with antagonistic radiopeptides that show slow internalization by cancer cells and low renal uptake. Still, comparison of ^64^Cu-labeled NOTA- and DOTA-conjugated agonistic bombesin analogs reveals lower tumor and renal radioactivity retention when using NOTA [24], which is in agreement with our present data. Very interesting is an evaluation of ^64^Cu-labeled A20FMDV2 peptide conjugates for imaging the integrin *α*_*v*_*β*_6_, since radiometal-labeled A20FMDV2 has high renal uptake [42]. In that study, the distribution of radioactivity after injection of ^64^Cu-NOTA-A20FMDV2 had the same features as distribution after injection of ^64^Cu-NOTA-ZHER2:S1, that is, rapid release of radioactivity from kidneys, poor retention of radioactivity in tumors, and an increase in hepatic uptake between 1 and 4 hours after injection, with a subsequent decrease at 24 h. Overall, increasing the time between ^64^Cu-NOTA-ZHER2:S1 or ^64^Cu-NODAGA-ZHER2:S1 injection and imaging did not improve imaging contrast and did not show any advantage over the use of ^68^Ga-NODAGA-ZHER2:S1.

It remains to be evaluated if the same phenomenon is relevant to all affibody molecules and not only to the HER2-targeting ones. However, the high renal reabsorption is typical for all tested radiometal-labeled affibody molecules, including EGFR-, HER3-, PDGFP*β*-, and IGFR-1R-specific molecules [30, 43–45]. In all cases, specificity tests based on presaturation of receptors in tumors did not decrease renal uptake although the probes were selected to have high affinity to murine counterparts of the human molecular targets. It is likely that this high renal reabsorption is caused by the high affinity of scavenger receptors in the kidneys to the affibody scaffold and is not dependent on target specificity. Thus, the results of this study are, most likely, relevant to all affibody molecules. Moreover, taken into account that all reabsorbed proteins and peptides are directed to the lysosomal compartment of tubuli cells [46], this effect might take place also for other scaffold proteins with high renal reabsorption. This assumption is supported by data concerning biodistribution of ^64^Cu-NOTA-A20FMDV2 [42], which has a high renal reabsorption and demonstrates a biodistribution pattern similar to the pattern of ^64^Cu-NOTA-ZHER2:S1. A number of alternative chelators for radiocopper have been suggested, such as cross-bridged cyclam derivatives [47, 48] or derivatives of sarcophagine [49, 50]. It should be evaluated if these chelators are better alternatives for labeling of scaffold proteins with ^64^Cu or ^61^Cu.

## 5. Conclusion

Our present results suggest that the molecular design of probes based on scaffold proteins with high renal reabsorption should avoid the combination of radiocopper and NOTA/NODAGA amido derivatives. This information is essential to the development of imaging probes based on DARPins, ADAPTs, and fibronectin domains, which have very high renal reabsorption, similar to that of affibody molecules [9, 11, 12].

## Figures and Tables

**Table 1:** Labeling of NOTA-ZHER2:S1 and NODAGA-ZHER2:S1 with ^64^Cu and conjugates stability under 2-hour challenge with a 500-fold excess of Na_4_EDTA.

	Overall yield^*∗*^ (%)	Purity (%)	Stability (% of affibody-associated activity)	
			Challenge	Control
	Protocol A			
^64^Cu-NOTA-ZHER2:S1	96.2	96.2	90.1 ± 1.2	96 ± 0.2
^64^Cu-NODAGA-ZHER2:S1	96.9	96.9	90.3 ± 0.7	96.1 ± 0.0

	Protocol B			
^64^Cu-NOTA-ZHER2:S1	86.1 ± 0.6	98.4 ± 0.5	99.1 ± 0.0	98.8 ± 0.1
^64^Cu-NODAGA-ZHER2:S1	86.6 ± 1.1	98.6 ± 0.7	98.5 ± 0.0	98.7 ± 0.4

^*∗*^Overall yield is defined as percentage of radionuclide incorporated into affibody molecules at the end of synthesis (Protocol A) or percentage of radionuclide incorporated in affibody molecules at the end of separation (Protocol B) (decay corrected).

**Table 2:** ^64^Cu-NOTA-ZHER2:S1 and ^64^Cu-NODAGA-ZHER2:S1 biodistribution in mice bearing HER2-positive (SKOV-3) xenografts.

	Uptake (% ID/g)					
	2 h		6 h		24 h	
	^64^Cu-NOTA- ZHER2:S1	^64^Cu-NODAGA- ZHER2:S1	^64^Cu-NOTA- ZHER2:S1	^64^Cu-NODAGA- ZHER2:S1	^64^Cu-NOTA- ZHER2:S1	^64^Cu-NODAGA- ZHER2:S1
Blood	1.0 ± 0.2	0.32 ± 0.05^*∗*^	1.4 ± 0.2	0.26 ± 0.04^*∗*^	1.15 ± 0.08	0.5 ± 0.1^*∗*^
Lung	3.0 ± 0.5	0.8 ± 0.1^*∗*^	5.9 ± 0.6	1.1 ± 0.2^*∗*^	4.7 ± 1.0	2.0 ± 0.6^*∗*^
Liver	10.9 ± 0.6	2.8 ± 0.5^*∗*^	17 ± 3	3.6 ± 0.5^*∗*^	11.3 ± 0.9	5 ± 1^*∗*^
Spleen	1.6 ± 0.2	0.56 ± 0.09^*∗*^	2.6 ± 0.7	0.63 ± 0.09^*∗*^	3.1 ± 0.5	1.2 ± 0.3^*∗*^
Stomach	3.3 ± 0.3	0.8 ± 0.1^*∗*^	5.2 ± 0.3	1.1 ± 0.2^*∗*^	3.5 ± 0.4	1.6 ± 0.4^*∗*^
Kidney	207 ± 35	290 ± 35^*∗*^	69 ± 7	226 ± 41^*∗*^	14 ± 1	110 ± 25^*∗*^
Tumor	17 ± 2	12 ± 3^*∗*^	17 ± 4	10 ± 2^*∗*^	10 ± 1	11 ± 4^*∗*^
Muscle	0.45 ± 0.06	0.16 ± 0.05^*∗*^	0.55 ± 0.09	0.14 ± 0.03^*∗*^	0.55 ± 0.04	0.25 ± 0.05^*∗*^
Bone	1.0 ± 0.1	0.35 ± 0.07^*∗*^	1.16 ± 0.05	0.30 ± 0.05^*∗*^	1.1 ± 0.2	0.5 ± 0.2^*∗*^

Data are presented as mean ± SD for four mice

^*∗*^Significant difference between ^4^Cu-NOTA-ZHER2:S1 and ^64^Cu-NODAGA-ZHER2:S1 at the given time point.

**Table 3:** Comparison of ^64^Cu-NOTA-ZHER2:S1 and ^64^Cu-NODAGA-ZHER2:S1 tumor-to-organ ratios in nude mice bearing SKOV-3 xenografts. Data are presented as mean ± SD for four mice.

	Tumor-to-organ ratio					
	2 h		6 h		24 h	
	^64^Cu-NOTA- ZHER2:S1	^64^Cu-NODAGA- ZHER2:S1	^64^Cu-NOTA-ZHER2:342	^64^Cu-NODAGA- ZHER2:S1	^64^Cu-NOTA-ZHER2:342	^64^Cu-NODAGA-ZHER2:342
Blood	17 ± 2	38 ± 5	12 ± 2	36 ± 4	8 ± 2	21 ± 8
Lung	5.6 ± 0.4	15 ± 2	2.8 ± 0.5	9 ± 2	2.1 ± 0.5	5 ± 1
Liver	1.6 ± 0.2	4.4 ± 0.8	1.0 ± 0.1	2.6 ± 0.4	0.8 ± 0.1	2.1 ± 0.8
Spleen	10.4 ± 0.7	22 ± 2	6.5 ± 0.6	15 ± 3	3.2 ± 0.6	9 ± 3
Stomach	5.1 ± 0.5	15 ± 2	3.2 ± 0.8	8 ± 1	2.8 ± 0.7	7 ± 3
Kidney	0.08 ± 0.01	0.04 ± 0.01	0.24 ± 0.04	0.042 ± 0.007	0.7 ± 0.1	0.10 ± 0.04
Muscle	38 ± 1	78 ± 10	30 ± 4	70 ± 14	17 ± 3	43 ± 13
Bone	18 ± 2	35 ± 3	14 ± 3	32 ± 9	9 ± 3	23 ± 7

The values for ^64^Cu-NOTA-ZHER2:S1 and ^64^Cu-NODAGA-ZHER2:S1 significantly differed for each tissue at all time points.

**Table 4:** Biodistribution of ^64^Cu-citrate in BALB/C Nu/Nu mice at two hours postinjection.

	Uptake	
	Per gram (% ID/g)	Per organ (% ID)
Blood	2.1 ± 0.1	
Lung	11 ± 1	1.6 ± 0.2
Liver	36.9 ± 0.5	31 ± 1
Spleen	3.2 ± 0.3	0.24 ± 0.04
Stomach	12.8 ± 0.8	1.29 ± 0.06
Kidney	14.1 ± 0.2	3.44 ± 0.07
Muscle	1.1 ± 0.1	
Bone	1.8 ± 0.3	
GI tract^*∗*^		28 ± 1
Carcass		29 ± 1
Totally		92 ± 5

Data are presented as mean ± SD for three mice.

^*∗*^The data for the GI tract (with contents) and carcass are presented for the whole sample.
